# Antipleuritic and Vascular Permeability Inhibition of the Ethyl Acetate-Petroleum Ether Stem Bark Extract of* Maerua angolensis* DC (Capparaceae) in Murine

**DOI:** 10.1155/2018/6123094

**Published:** 2018-07-11

**Authors:** Felix Agyei Ampadu, Eric Boakye-Gyasi, Newman Osafo, Charles Kwaku Benneh, Edmund Ekuadzi, Eric Woode

**Affiliations:** ^1^Department of Pharmacology and Toxicology, School of Pharmacy, Central University, Miotso, Ghana; ^2^Department of Pharmacology, Faculty of Pharmacy and Pharmaceutical Sciences, College of Health Sciences, Kwame Nkrumah University of Science and Technology, Kumasi, Ghana; ^3^Department of Pharmacology and Toxicology, School of Pharmacy, University of Health and Allied Sciences, Ho, Ghana; ^4^Department of Pharmacognosy, Faculty of Pharmacy and Pharmaceutical Sciences, College of Health Sciences, Kwame Nkrumah University of Science and Technology, Kumasi, Ghana

## Abstract

*Maerua angolensis* has been used traditionally in the management of pain, arthritis, and rheumatism in Ghana and Nigeria but no scientific evidence is currently available to give credence to its folkloric use. The aim of this study was therefore to evaluate the anti-inflammatory effects of a stem bark extract of* Maerua angolensis *DC (MAE) in acute inflammatory models. The effects of MAE (30-300 mg kg^−1^) on neutrophil infiltration, exudate volume, and endogenous antioxidant enzymes in lung tissues and lung morphology were evaluated with the carrageenan induced pleurisy model in Sprague Dawley rats. The effects of MAE (30-300 mg kg^−1^) on vascular permeability were also evaluated in the acetic acid induced vascular permeability in ICR mice. MAE significantly reduced neutrophil infiltration, exudate volume, and lung tissue damage in carrageenan induced pleurisy. MAE increased the activities of antioxidant enzymes glutathione, superoxide dismutase, and catalase in lung tissues. The extract was also able to reduce myeloperoxidase activity and lipid peroxidation in lung tissues in carrageenan induced rat pleurisy. Vascular permeability was also attenuated by the extract with marked reduction of Evans blue dye leakage in acetic acid induced permeability assay. The results indicated that* Maerua angolensis* is effective in ameliorating inflammation induced by carrageenan and acetic acid. It also has the potential of increasing the activity of endogenous antioxidant enzymes.

## 1. Introduction

Inflammation is a defensive response to injury to curb further damage and to also initiate tissue repair [1]. It is currently understood that inflammation is part of the nonspecific immune response that occurs in reaction to any type of cellular injury which may be mechanical (e.g., contusion or abrasion), chemical (e.g., toxins, acid, and alkaline), physical (e.g., extreme heat or cold), microbes (e.g., bacteria, virus, and parasites), necrotic tissue, oxidative stress, and/or immunological reactions. In the inflammatory response there are increased blood flow, cellular metabolism, vasodilatation, release of soluble mediators, fluid extravasation, and cellular influx. Under normal conditions, inflammation is self-limiting but in some disease states there is persistent injury and further cell damage which then leads to chronic inflammatory disorders [2, 3]

*Maerua angolensis* is a tropical plant that is widespread in the savannah area of tropical Africa to South Africa and Swaziland [4].* Maerua angolensis* has a long history of use in traditional medicine to manage various conditions such as psychosis, epilepsy, pain, gout, diabetes, peptic ulcer, diarrhea, and arthritis in Nigeria and other West African countries [5, 6]. Most of the medicinal plants from folklore have not been assessed for their toxicity, mechanisms of action, and interactions with food and other medicines [7]. Hence, it is prudent to conduct further studies into medicinal plants to obtain new data on indications and safety profile.

The cellular and molecular mechanism of the carrageenan induced pleurisy is well characterized [8]. The early phase of the carrageenan induced inflammation is related to the production of histamine, leukotrienes, platelet-activating factor, and possibly cyclooxygenase products, while the delayed phase of the carrageenan-induced inflammatory response has been linked to neutrophil infiltration and the production of neutrophil-derived free radicals and oxidants, such as hydrogen peroxide, superoxide, and hydroxyl radical, as well as to the release of other neutrophil-derived mediators [9, 10] that causes tissue damage. As neutrophils play major roles in acute and chronic inflammation, identification of compounds that inhibit its infiltration to the site of inflammation may result in new paradigms in the management of inflammatory diseases [11, 12]. The carrageenan pleurisy model allows the assessment and quantification of multiple inflammatory parameters. After vasodilation in the initial phase inflammation endothelial cells in local capillary beds contract, generating spaces between the cells which substantially increase vascular permeability. Vascular permeability enhances fluid and cellular extravasation which results in localized edema observed in the inflammatory response. It has been well established that the intraperitoneal injection of acetic acid greatly enhances vascular permeability and facilitates the vascular leakage of Evans blue dye [13]. The use of Evans blue dye as an* in vivo* marker through vascular permeability facilitates the investigation of the effect of pathological changes in various disorders mainly, immunological disorders, inflammatory disorders, and cardiovascular diseases like atherosclerosis myocardial infarction, cancers, and others.

## 2. Methods

### 2.1. Plant Collection and FT-IR Analysis of Extract

The stem bark of* Maerua angolensis* was collected from the rocky areas of Kwahu Tafo to Nkyenenkyene road, Eastern Region, Ghana (6.415360 N 0.363160 W), and was authenticated by Professor Kofi Annan, Department of Herbal Medicine, Faculty of Pharmacy and Pharmaceutical Sciences, KNUST, Kumasi. A voucher specimen (KNUST/FP/12/051) was deposited at the herbarium at the faculty.

The stem bark was room-dried for seven days and pulverized into fine powder. The powder was extracted with a mixture of petroleum ether-ethyl acetate (1:1) at 25°C to obtain an extract labelled as MAE throughout this study. The extract was then concentrated under reduced pressure at 60°C using a rotary evaporator (Rotavapor R-215, BÜCHI Labortechnik AG, Flawil, Switzerland) into green viscous mass. It was further dried in a hot air oven at 50°C for one week and then kept in a freezer (-20°C) until use (yield = 0.47 % w/w).

**Figure 1:**
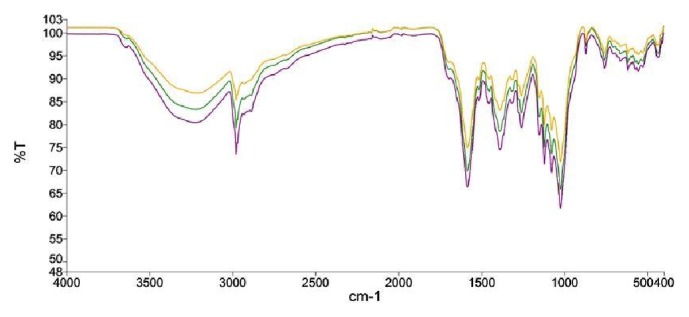
Baseline corrected infrared spectra of MAE. Experiment repeated thrice, with similar conditions, from 400 to 4000 cm^−1^.

In order to identify possible functional groups that may be present in the extract, a triplicate FT-IR (PerkinElmer UATR Two) spectum was generated and baseline corrected. The spectra between 400–1400 cm^−1^ are usually considered as the unique region for every compound/compound mixtures and hence can be used for identification and quality control. The characteristic spectra (Figure 1) in the region from 400-1400 cm^−1^ were as a fingerprint region for subsequent comparison of future extracts.

### 2.2. Animals

Sprague Dawley rats (200–220 g) and Imprint Control Region (ICR) mice (20-30 g) were procured from Noguchi Memorial Institute for Medical Research, University of Ghana, Legon, and housed in the animal facility of the Department of Pharmacology, KNUST, Kumasi, Ghana. The animals were kept in stainless steel cages, with wood shavings as bedding, fed with normal pellet diet and water available* ad libitum*. Sample size of 5-6 animals per group was utilized throughout the study, following guidelines according to National Institute of Health Guidelines for the Care and Use of Laboratory Animals (NIH, Department of Health and Human Services publication no. 85-23, revised 1985). All procedures used in this study were approved by the Department of Pharmacology Ethics Committee, College of Health Sciences (CoHS), KNUST, Kumasi, Ghana.

### 2.3. Chemicals and Reagents

 Diclofenac sodium was purchased from Troge Medical GmbH, Hamburg, Germany. Phosphate buffered saline (PBS) was obtained from Gibco, Karlsruhe, Germany; methanol, ethanol, and sodium bicarbonate were obtained from Fisher Scientific, UK; acetic acid was obtained from BDH, Poole, England; trichloroacetic acid and haematoxylin and eosin Y were obtained from Abbey Color, Philadelphia, USA; xylene, formaldehyde, thiobarbituric acid, EDTA, monopotassium dihydrogen phosphate, dipotassium monohydrogen phosphate, monosodium phosphate, disodium phosphate, *λ*-carrageenan, o-dianisidine dihydrochloride, and Evans blue dye were obtained from Sigma-Aldrich, St. Louis, MO, USA.

### 2.4. Carrageenan Induced Rat Pleurisy

Rats (200-220 g) (n=6) were anesthetized with ether (0.25 mL in a 3 L container) open-drop method and subjected to a skin incision at the left sixth intercostal space. The underlying muscle was cut open, and 1% w/v *λ*-carrageenan suspension (0.1 mL) in normal saline was injected into the pleural space [14, 15]. The skin opening was closed with a stitch, and the rats were allowed to recover. The extract MAE (30, 100, and 300 mg kg^−1^,* p.o.*) or vehicle (10 mL kg^−1^,* p.o*.) and diclofenac (10, 30, and 100 mg kg^−1^, i.p.) were given 1 h and 30 min, respectively, before the injection of carrageenan. The rats were then sacrificed with excess of ether 6 h after carrageenan injection. The pleural cavity was opened and washed with 2 mL of saline solution containing 0.1% of EDTA. The pleural fluids were then aspirated and the volumes quantified (mL). The actual exudate volume was determined by subtracting the volume of solution injected (2 mL) from the total volume of fluid aspirated. Pleural fluids tainted with blood were excluded. The mobilized neutrophils in the exudate were quantified using an automated analyzer (ABX micros 60-Horiba, Irvine (CA), USA).

### 2.5. Histological Examination

Lung biopsies were taken 6 h after injection of carrageenan. Lung sections were collected and stored in 10% buffered formalin. The lung tissues were dehydrated with graded ethanol, embedded in paraffin, blocked, and sectioned. The sections were stained with hematoxylin and eosin and examined under light microscopy [16] (Axioscope Zeiss Microscope, Carl Zeiss Microimaging, Heidelberg, Germany). Damage to lung tissues was assessed by light microscopy [17]. In each treatment group, six random fields of view were analyzed by observers unaware of the treatment protocols. The degree of microscopic lung damage induced by carrageenan was assessed. Histological slides were scored according to the following parameters: hyperaemia, oedema, alveolar septal thickening, and neutrophil infiltration [18]. The degree of the disorganization was quantified on a scale of 0–4 (i.e., 0: not present, 1: very mild, 2: mild, 3: moderate, and 4: extensive).

### 2.6. Myeloperoxidase (MPO) Activity

Myeloperoxidase activity is an index of neutrophil accumulation. The influence of MAE on MPO activity in lung tissues was measured according to the method described by Bradley et al. [19, 20]. The assay mixture consisted of 0.3 mL 0.1 M phosphate buffer (pH 6.0), 0.3 mL 0.01 M H_2_O_2_, 0.5 mL 0.02 M o-dianisidine (freshly prepared) in deionized water, and 10 *μ*L lung homogenate supernatant in a final volume of 3.0 mL. The supernatant was added last and the change in absorbance at 460 nm was monitored every 1 min for 10 min with a microplate reader (Synergy H1 Multi-Mode plate reader, Winooski, VT, USA). All measurements were carried out in triplicate. MPO activity was explained as one unit that increases absorbance at a rate of 0.001 min^−1^ and specific activity was expressed as units/mg protein.

### 2.7. Glutathione Assay

Aliquots 0.1 mL of 10 % tissue homogenate were mixed with 2.4 mL of 0.02 M EDTA solution and kept on ice bath for 10 min. Then 2 mL of distilled water and 0.5 mL of trichloroacetic acid (TCA) 50 % (w/v) were centrifuged at 3000 × g for 20 min at 4°C to remove precipitate. The supernatants, 1 mL, were then mixed with 2.0 mL of Tris buffer (0.4 M, pH 8.9) and 0.05 mL of 5'-dithiobisnitrobenzoic acid (DTNB) solution; Ellman's reagent (10 mM) was added and swirled carefully. The absorbance was measured at 412 nm against a reagent blank with no homogenate after addition of DTNB and incubation at room temperature for 5 minutes [21, 22]. Glutathione was then quantified from a glutathione standard curve and expressed as *μ*M mg^−1^ of protein.

### 2.8. Catalase Activity (CAT)

Catalase activity was measured as described by Aebi [23, 24]. It was determined by measuring the reduction in hydrogen peroxide (20 s interval) concentration at 240 nm for 60 s.

Medium consists of 130 *μ*L 50 mM potassium buffer (pH 7.0) and enzyme extract, 65 *μ*L of 10 mM H_2_O_2_. The blank had 65 *μ*L of the potassium phosphate and 130 *μ*L of sample. The concentration of H_2_O_2_ was estimated from the absorbance using the following equation:

(1) $$\begin{array}{c} \left[\;H_{2}O_{2}\;\;mM\;\right]=\frac{{Absorbance}_{240\;\;nm}x\;\;1000}{39.4\;\;{mol}^{-1}{cm}^{-1}} \end{array}$$

where 39.4 mol^−1^cm^−1^ is the molar extinction coefficient for H_2_O_2_. CAT activity was expressed as U mg^−1^ protein.

### 2.9. Superoxide Dismutase Activity (SOD)

SOD activity was determined as described by Misra and Fridovich [25, 26]. It is based on the ability of SOD to inhibit autoxidation of adrenaline to adrenochrome. 0.5 mL of tissue homogenate, 0.75 mL of ethanol, and 0.15 mL of chloroform (4°C) were combined in mixture. The mixture was then centrifuged at 2000 rpm for 20 min. Subsequently, 0.5 mL of supernatant, 0.5 mL of 0.6 mM EDTA solution, and 1 mL of carbonate bicarbonate buffer (0.1 M, pH 10.2) were added. The reaction was commenced by adding 0.05 mL of 1.3 mM adrenaline and the increase in absorbance at 480 nm due to the adrenochrome formation was measured with a microplate reader (Synergy H1 Multi-Mode plate reader, Winooski, VT, USA). One unit of SOD activity was defined as the amount of protein causing 50% inhibition of the autoxidation of adrenaline at 25°C.

(2) %  inhibition=Absorbancetest−AbsorbancereferenceAbsorbancetest  x  100Units  of  activity  per  mg  protein=%  inhibition50  x  weight  of  protein

### 2.10. Malondialdehyde Measurement (MDA)

Lipid peroxidation is an important marker of oxidative stress in pleuritis. Carrageenan induced pleurisy and lipid peroxidation (as analyzed by MDA) have been previously associated [27]. The extent of lipid peroxidation in lung tissues was assessed by measuring MDA as described by Heath and Packer [28, 29]. 3 mL of 20% trichloroacetic acid containing 0.5% thiobarbituric acid was added to a 1 mL aliquot of lung homogenate supernatant in a test tube.

The mixture obtained was heated in a water bath (95°C) for 30 min and then allowed to cool. The test tube was then centrifuged at 10,000×g for 10 min, and the absorbance of the supernatant at 532 nm was measured. The value for the nonspecific absorption at 600 nm was subtracted from the 532 nm reading. The concentration of MDA was calculated using MDA's extinction coefficient of 155 mM^−1^cm^−1^.

### 2.11. Acetic Acid Induced Vascular Permeability

The acetic acid-induced vascular permeability test with slight modifications was conducted as previously described by Whittle [30, 31]. Groups of mice (25-30 g) (n=6) were treated with either MAE (30, 100, and 300 mg kg^−1^,* p.o*.), diclofenac (30 mg kg^−1^,* i.p*.), or vehicle. 1 h after treatments, each mouse was injected intravenously with 2 % Evan's blue solution at 0.1 mL 10 g^−1^ body weight through the tail vein. 10 min afterwards, each mouse received 0.6 % acetic acid solution intraperitoneally at 0.1 mL 10 g^−1^ body weight. 30 min after acetic acid injection, the mice were sacrificed and peritoneal cavity washed three times with saline (10 mL). Saline washes were centrifuged for 5 min at 3500 rpm. The supernatants were collected and their absorbance was measured at 590 nm with a plate reader (Synergy H1 Multi-Mode plate reader, Winooski, VT, USA). Evans blue extravasation was enumerated from a standard curve and expressed in *μ*g.

### 2.12. Statistical Analysis

The experimental data was expressed as mean ± SEM. Significance of difference among various treated groups and control group was analyzed by means of one-way analysis of variance followed by Dunnett's multiple comparison test with GraphPad Prism for Windows version 5.01 (GraphPad Software, San Diego, CA, USA). P value less than 0.05 was considered significant.

## 3. Results

### 3.1. Effects of Extract on Carrageenan Induced Pleurisy

**Figure 2:**
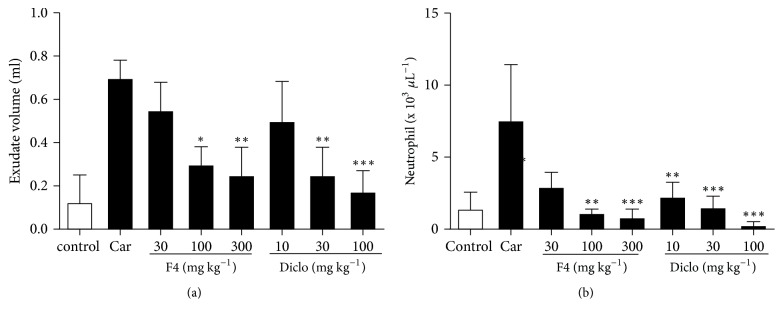
Effects of MAE (30-300 mg kg^−1^* p.o.*) and diclofenac (10-100 mg kg^−1^* i.p.*) on exudate volume (a) and neutrophil accumulation (b) in the pleural cavity were assessed 6 h after carrageenan injection. Data are expressed as mean ± SEM. n=5, ^*∗*^*P*<0.05, ^*∗∗*^*P*<0.01, and ^*∗∗∗*^*P*<0.001 vs. carrageenan only treated group (one-way ANOVA followed by Dunnett's Multiple Comparison test).

Injection of carrageenan into the pleural cavity of rats elicited an inflammatory response within 6 h, characterized by the accumulation of fluid that contained large numbers of inflammatory cells as shown in Figure 2. MAE (300 mg kg^−1^) significantly inhibited the inflammatory response decreasing of exudate formation (F_7,20_=10.84,* P*<0.0001) and neutrophil infiltration (F_7,20_=8.86,* P*<0.0001) with a maximal effect of 64.2% and 90.9%, respectively (Figure 2).

MAE (100 mg kg^−1^) also significantly reduced exudate formation (F_7,20_=9.58,* P*<0.0001) and neutrophil infiltration (F_7,20_=8.41,* P*<0.0001) with a maximal effect at 57.1% and 86.5%, respectively. Diclofenac (100 mg kg^−1^) used as a standard anti-inflammatory agent also significantly reduced exudate formation (F_7,20_=14.96,* P*<0.0001) and neutrophil infiltration (F_7,20_=9.18,* P*<0.0001) almost completely with a maximal effect of 75% and 96.6%, respectively (Figure 2).

**Figure 3:**
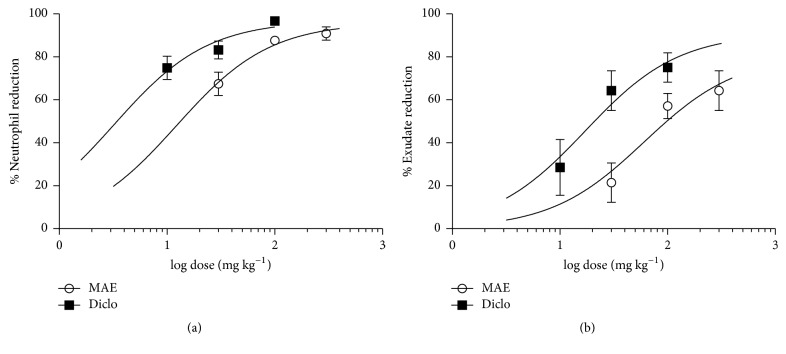
Dose response curves for diclofenac (10-100 mg kg^−1^* i.p.*) compared to MAE (30-300 mg kg^−1^* p.o.*) on neutrophil counts (a) and exudate volumes (b).

From the ED_50_ calculated from the dose response curves (Figures 3(a) and 3(b)), MAE was found to be approximately 3.8× less potent (ED_50_=12.26±3.90) than diclofenac (ED_50_=3.22±4.45) in reducing neutrophil infiltration and 3.5x less potent (ED_50_=60.57±15.82) than diclofenac (ED_50_=17.13±17.11) in reducing exudate formation.

### 3.2. Histopathology

**Figure 4:**
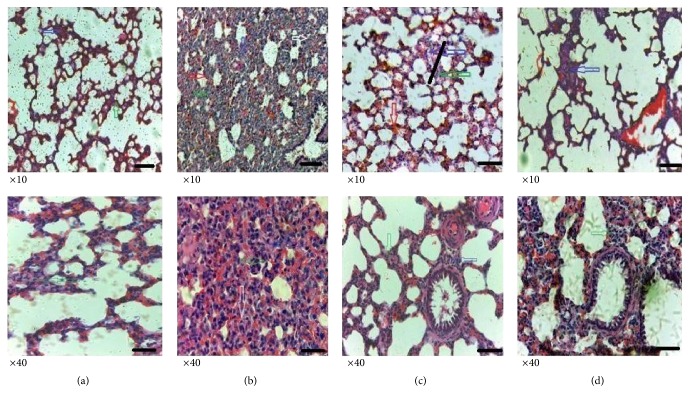
Histopathological study in carrageenan induced pleurisy in rats. Histological slides showing vehicle only (a), vehicle + carrageenan (b), MAE (300 mg kg^−1^) + carrageenan (c), and diclofenac (100 mg kg^−1^) + carrageenan (d). Micron bar represents 100 *μ*m.

**Figure 5:**
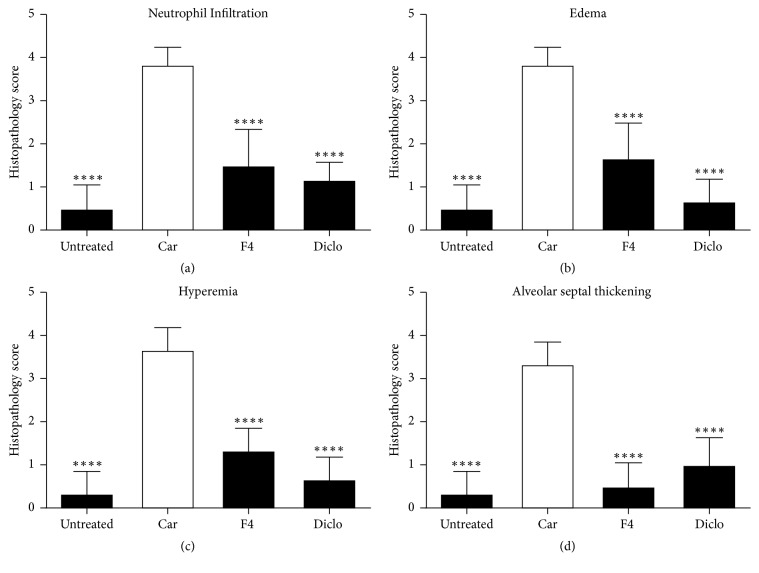
Histological scoring of lung injury in carrageenan induced pleurisy. MAE and diclofenac reduced lung injury significantly based on the following scoring indices: neutrophil infiltration (a) (^*∗∗∗∗*^*P*<0.0001), edema (b) (^*∗∗∗∗*^*P*<0.0001), hyperemia (c) (^*∗∗∗∗*^*P*<0.0001), and alveolar septal thickening (d) (^*∗∗∗∗*^*P*<0.0001) compared to carrageenan only treated groups. The degree of the lung damage was scored on a scale of 0–4. (i.e., 0: not present, 1: very mild, 2: mild, 3: moderate, and 4: extensive).

Histological examinations aid in the identification and assessment of microscopic morphological changes in cells and tissues. Vehicle only treated rats showed normal lung architecture (Figure 4(a)) with little or no signs of neutrophil infiltration, oedema, hyperaemia, and alveolar septal thickening. Carrageenan treated rats that received no drug treatment showed extensive disorganization of alveolar structures (Figure 4(b)) with significant presence of neutrophils, oedema, hyperaemia, and alveolar septal thickening (Figure 5). When rats were treated with MAE (300 mg kg^−1^), there was reduced alveolar structural disorganization (Figure 4(d)). This presented with a significantly (*P*<0.0001) suppressed neutrophil infiltration, oedema, hyperaemia, and alveolar septal thickening (Figure 5).

Rats treated with a diclofenac and carrageenan (Figure 4(c)) showed significant (*P*<0.0001) suppression of neutrophil infiltration with minor or no oedema, hyperaemia, and alveolar septal thickening when compared with carrageenan only treated rats (Figure 5).

### 3.3. Enzyme Assays

**Figure 6:**
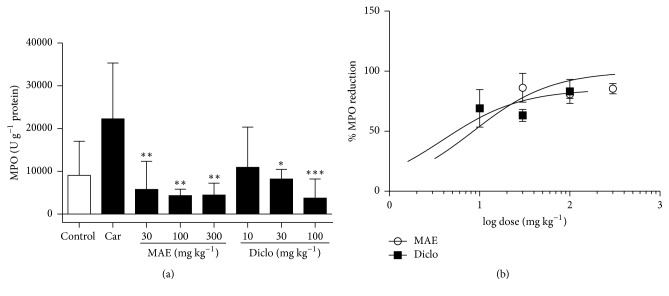
Effects of MAE (30-300 mg kg^−1^* p.o.*) and diclofenac (10-100 mg kg^−1^* i.p.*) on MPO activity were assessed from lung tissue 6 h after carrageenan injection. Data are expressed as mean ± SEM. n=5, ^*∗*^*P*<0.05, ^*∗∗*^*P*<0.01, and ^*∗∗∗*^*P*<0.001 vs. carrageenan only treated group (one-way ANOVA followed by Dunnett's Multiple Comparison test) (a). Dose response curves for diclofenac (10-100 mg kg^−1^* i.p.*) compared to MAE (30-300 mg kg^−1^* p.o.*) on MPO activity (b).

MPO and CAT activity and MDA level in the lung tissue were increased after intrapleural injection of carrageenan. Compared to carrageenan only treated tissues, there was a significant (*P*<0.05) reduction in MPO activity in MAE (30, 100, and 300 mg kg^−1^) treated rats. Levels of MDA also reduced significantly in MAE (100 and 300 mg kg^−1^) treated rats. MAE also increased CAT activity significantly at all doses (Figures 6(a), 9(a), and 10(a)). Similar reductions in MPO activity and MDA levels were observed at 30 and 100 mg kg^−1^ of diclofenac.

Additionally, catalase activity was significantly increased at all tested doses ((10-100 mg kg^−1^) of diclofenac. From the dose response analysis, the potency ratios of diclofenac and MAE with respect to MPO activity, CAT activity, and MDA expression are 2.1, 12.8, and 1.2, respectively.

**Figure 7:**
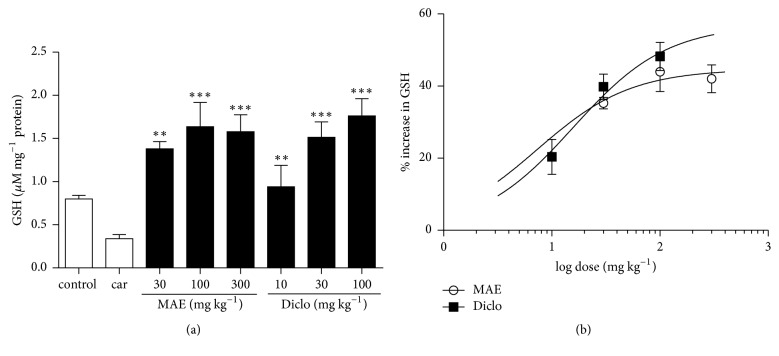
Effects of MAE (30-300 mg kg^−1^* p.o.*) and diclofenac (10-100 mg kg^−1^* i.p.*) on GSH levels were assessed from lung tissue 6 h after carrageenan injection. Data are expressed as mean ± SEM. n=5, ^*∗∗*^*P*<0.01, and ^*∗∗∗*^*P*<0.001 vs. carrageenan only treated group (one-way ANOVA followed by Dunnett's Multiple Comparison test) (a). Dose response curves for diclofenac (10-100 mg kg^−1^* i.p.*) and MAE (30-300 mg kg^−1^* p.o.*) on GSH levels (b).

**Figure 8:**
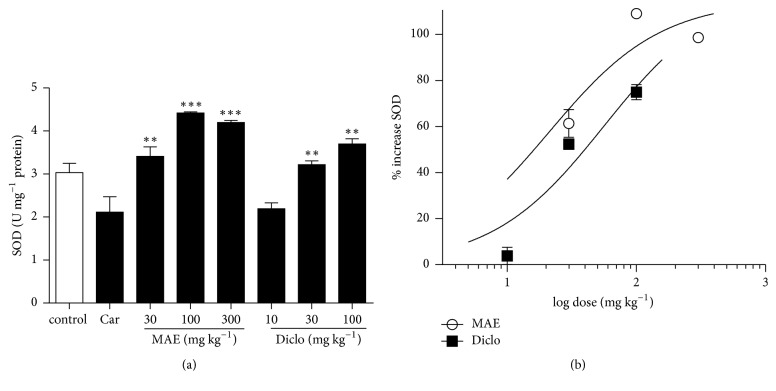
Effects of MAE (30-300 mg kg^−1^* p.o.*) and diclofenac (10-100 mg kg^−1^* i.p.*) on SOD activity were assessed from lung tissue 6 h after carrageenan injection. Data are expressed as mean ± SEM. n=5, ^*∗∗*^*P*<0.01, and ^*∗∗∗*^*P*<0.001 vs. carrageenan only treated group (one-way ANOVA followed by Dunnett's Multiple Comparison test) (a). Dose response curves for diclofenac (10-100 mg kg^−1^* i.p.*) and MAE (30-300 mg kg^−1^* p.o.*) on SOD activity (b).

**Figure 9:**
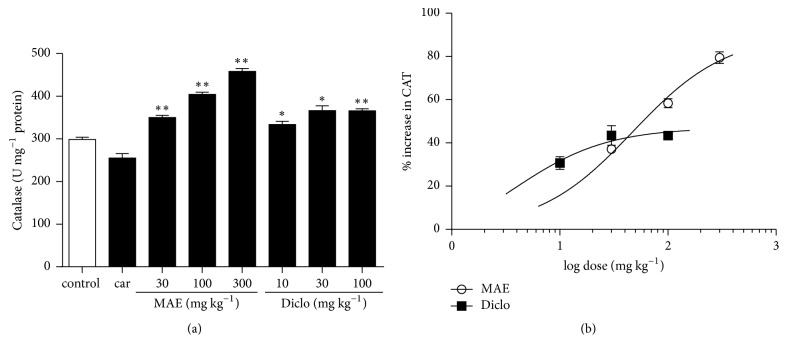
Effects of MAE (30-300 mg kg^−1^* p.o.*) and diclofenac (10-100 mg kg^−1^* i.p.*) on catalase activity were assessed from lung tissue 6 h after carrageenan injection. Data are expressed as mean ± SEM. n=5, ^*∗*^*P*<0.05, and ^*∗∗*^*P*<0.01 vs. carrageenan only treated group (one-way ANOVA followed by Dunnett's Multiple Comparison test) (a). Dose response curves for diclofenac (10-100 mg kg^−1^* i.p.*) and MAE (30-300 mg kg^−1^* p.o.*) on catalase activity (b).

**Figure 10:**
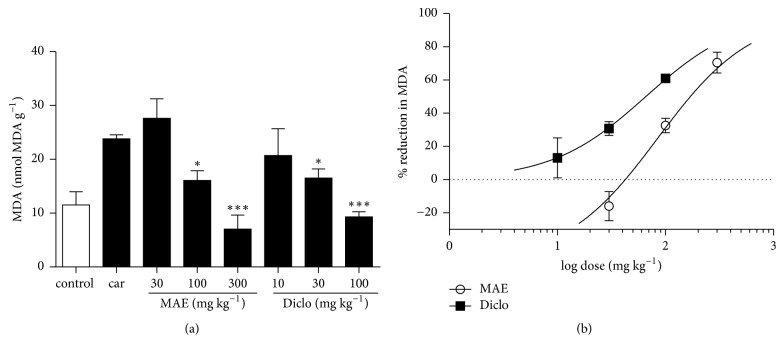
Effects of MAE (30-300 mg kg^−1^* p.o.*) and diclofenac (10-100 mg kg^−1^* i.p.*) on MDA levels were assessed from lung tissue 6 h after carrageenan injection. Data are expressed as mean ± SEM. n=5, ^*∗*^*P*<0.05, and ^*∗∗∗*^*P*<0.001 vs. carrageenan only treated group (one-way ANOVA followed by Dunnett's Multiple Comparison test) (a). Dose response curves for diclofenac (10-100 mg kg-1* i.p*) and MAE (30-300 mg kg^−1^* p.o.*) on MDA levels (b).

GSH and SOD present in vehicle only treated tissues significantly decreased after carrageenan injection. Compared to carrageenan only treated lung tissues, there was a significant increase in GSH levels and SOD activity in 30, 100, and 300 mg kg^−1^ MAE (*P*<0.0001) treated lung tissues (Figures 7(a) and 8(a)). Similarly, the positive control diclofenac also showed significant increase in GSH levels and SOD activity at all doses (10-100 mg kg^−1^) compared to carrageenan only treated tissues (*P*<0.0001) (Figures 7(a) and 8(a)). From the ED_50_ calculated from the dose response curves (Figures 7(b) and 8(b)), MAE is approximately 2.4x more potent than diclofenac in increasing both GSH expression and SOD activity.

### 3.4. Vascular Permeability

**Figure 11:**
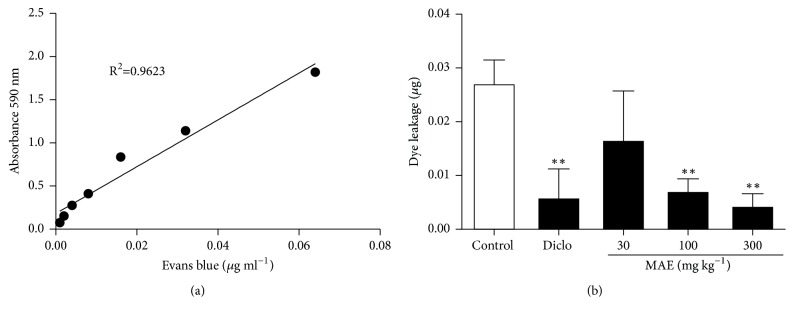
Evans blue dye standard curve (a) and effects of MAE (30-300 mg kg^−1^* p.o.*) and diclofenac (30 mg kg^−1^* i.p.*) on Evans blue dye extravasation into the peritoneal cavity of mice (b). Data are expressed as mean ± SEM. n=5; ^*∗∗*^*P*<0.01 vs. vehicle treated group. One-way ANOVA followed by Dunnett's Multiple Comparison test.

Injection of acetic acid into the peritoneum of mice previously injected intravenously with 2% Evans blue dye exhibited an inflammatory response after 30 min, characterized by the extravasation of Evans blue dye into the peritoneal cavity of the mice. The effect of MAE on dye extravasation in peritoneal fluid after acetic acid challenge was determined with an Evans blue standard curve as shown in Figure 11(a). There was a significant decrease in dye leakage in mice treated with MAE at 100 mg kg^−1^ (73% inhibition,* P*<0.005) and 300 mg kg^−1^ (83% inhibition,* P*<0.0025) when compared to controls. Diclofenac at a dose of 30 mg kg^−1^ also caused a significant decrease in dye leakage (78% inhibition,* P*<0.003) when compared to controls (Figure 11(b)).

## 4. Discussion

In this study, the anti-inflammatory properties of an ethyl acetate-petroleum ether (1:1) stem bark extract of* M. angolensis *(MAE) were evaluated. The carrageenan induced rat pleurisy model, which is useful for evaluating anti-inflammatory drug candidates, especially those derived from natural products and the acetic acid induced vascular permeability models were employed. This model is characterized by an early phase (4 h after carrageenan administration), in which leukocyte migration to the pleural cavity and the lungs is significantly enhanced and histological changes occurs [14, 30]. This early response is associated with a marked activation of the NF-*κ*B and p38 MAPK pathways, which leads to a massive generation of proinflammatory mediators such as TNF-*α*, IL-1*β*, and NO, in addition to the increased activity of important proinflammatory enzymes such as adenosine deaminase (ADA) and MPO [8, 32]

Cellular infiltration was observed when carrageenan was injected into the pleural cavity of rats. There was an inflammatory reaction associated with exudation of fluids into the pleural space accompanied by a high influx of neutrophils. This ultimately leads to the increased levels of prostaglandin E_2_, TNF-*α* and IL-1*β*, ROS, and lipid peroxidation [33]. Cell migration occurs as a consequence of several processes including adhesion and cell mobility [34].

MAE-treated groups showed significantly fewer neutrophils in the pleural exudates than the controls, suggesting the inhibition of neutrophil infiltration which may be due to inhibition of rolling and adhesion of neutrophils, which impaired neutrophil migration from blood vessels. MAE produced a dose dependent inhibition in the influx of neutrophils and exudate volume into the pleural cavity similar to diclofenac. PGE_2_ is the mediator primarily responsible for the exudation that occurs in carrageenan induced pleurisy via EP2 and EP3 receptors [35, 36].

Hence, inhibition of EP2 and EP3 receptors might contribute to the anti-inflammatory activity of MAE. Exudate formation also results from elevated NO levels produced by activation of inducible nitric oxide synthase (iNOS) in endothelial and inflammatory cells [37, 38]. Since MAE was able to reduce exudate levels, it may be involved in the blockade of iNOS activation. Additionally, the neutrophil decrease that was observed was followed by a reduction in MPO activity [39]. MPO is a proinflammatory enzyme found in cytoplasmic granules of neutrophils which leads to the generation of cytotoxic compounds such as hypochlorous acid and tyrosyl radicals from hydrogen peroxide and tyrosine, respectively [40, 41].

Oedema is just one component of the inflammatory response; increased vascular permeability also plays a significant role. Hence, to further investigate the inhibitory effect of the extract on the prostaglandin, serotonin, and histamine pathways of inflammation, the role of the extract in acetic acid induced vascular permeability was investigated. In acetic acid-induced vascular permeability test, acetic acid challenge brings about increases in the level of mediators such as prostaglandins, serotonin, and histamine in peritoneal fluids, which in turn lead to vasodilation and an increase in vascular permeability [13, 42]. MAE dose-dependently attenuated the capillary permeability induced by acetic acid in mice. These findings suggest that the anti-inflammatory effect of MAE on the acute phase of inflammation might be associated with prevention of vasodilation and inhibition of the release of inflammatory mediators such as histamine and serotonin.

In acute inflammation, there is decreased activity of endogenous antioxidant enzymes (CAT, SOD, and GSH) and increased lipid peroxidation in tissues as a result of oxidative damage [43]. CAT, SOD, and GSH also play a crucial role as protective enzymes. Schreck et al. [44] demonstrated that antioxidants inhibit nuclear factor kappa light-chain-enhancer of activated B cells (NF-kB), whereas ROS activate NF-*κ*B, a transcription factor which activates the transcription of several genes involved in inflammation [45, 46]. The protective role of GSH against inflammatory diseases has been proven by depleting endogenous GSH with Buthionine sulfoximine (BSO) [47, 48] which resulted in aggravating effect on various models of inflammation including carrageenan induced pleurisy. CAT which is localized in subcellular organelles of peroxisomes catalyses the conversion of hydrogen peroxide to water and oxygen [49]; SOD in cells work in conjunction with H_2_O_2_-removing enzymes such as glutathione peroxidase (GPx) or CAT to prevent action of H_2_O_2_, which in turn inhibits the formation of hydroxyl radicals [50, 51]. Treatment with MAE increased the activities of CAT, SOD, and GSH. The above effects resulted in decreased lipid peroxidation from the low levels of MDA in MAE-treated rats thereby significantly reducing the severity of inflammation.

Histopathology can offer a pronounced structural peculiarity as a pragmatic, univocal, and decisively characteristic sign of an inflammatory process [52]. Henceforth, histopathological studies on lung sections after carrageenan challenge were carried out which showed the extract was able to preserve normal alveolar architecture with reduced influx of neutrophils and oedema formation. The alveolar walls were markedly less thickened with less hyperaemia. MAE showed ability to attenuate lung injury induced by carrageenan and this is in agreement with Wilson et al. [53, 54] finding which established that materials that act on multiple proinflammatory mediators improve lung function in inflammation.

## 5. Conclusion

The ethyl acetate-petroleum ether stem bark extract of* Maerua angolensis* has anti-inflammatory activity in acute inflammation by attenuating carrageenan induced pleurisy and decreasing acetic acid-induced vascular permeability. The extract has also exhibited significant* in vivo* antioxidant activity, which may contribute to its anti-inflammatory activity.

## Data Availability

The data used to support the findings of this study are available from the corresponding author upon request.
